# Speciation trajectories in recombining bacterial species

**DOI:** 10.1371/journal.pcbi.1005640

**Published:** 2017-07-03

**Authors:** Pekka Marttinen, William P. Hanage

**Affiliations:** 1 Helsinki Institute for Information Technology HIIT, Department of Computer Science, Aalto University, Espoo, Finland; 2 Center for Communicable Disease Dynamics, Department of Epidemiology, Harvard TH Chan School of Public Health, Boston, MA, USA; University of New South Wales, AUSTRALIA

## Abstract

It is generally agreed that bacterial diversity can be classified into genetically and ecologically cohesive units, but what produces such variation is a topic of intensive research. Recombination may maintain coherent species of frequently recombining bacteria, but the emergence of distinct clusters within a recombining species, and the impact of habitat structure in this process are not well described, limiting our understanding of how new species are created. Here we present a model of bacterial evolution in overlapping habitat space. We show that the amount of habitat overlap determines the outcome for a pair of clusters, which may range from fast clonal divergence with little interaction between the clusters to a stationary population structure, where different clusters maintain an equilibrium distance between each other for an indefinite time. We fit our model to two data sets. In *Streptococcus pneumoniae*, we find a genomically and ecologically distinct subset, held at a relatively constant genetic distance from the majority of the population through frequent recombination with it, while in *Campylobacter jejuni*, we find a minority population we predict will continue to diverge at a higher rate. This approach may predict and define speciation trajectories in multiple bacterial species.

## Author summary

Species are conventionally defined as groups of individuals that breed with each other, but not with those of other species. However, this does not apply to bacteria because, even if they reproduce clonally, DNA may be donated between distinct species. Nevertheless, bacterial species do exist, and a fundamental question is how they are created. We present a mathematical model to describe bacterial speciation. The model predicts that two groups of ecologically different bacteria, assumed to live in partially overlapping habitats, may evolve into genetically distinguishable clusters, without being able to proceed to full separation. Analysis of a divergent *Streprococcus pneumoniae* subgroup shows that such ‘satellite species’ exist and can be distinguished from more rapidly diverging clusters, like the one we detect in *Campylobacter jejuni*.

## Introduction

Speciation in eukaryotes is well-studied [1], but the definition of bacterial species remains controversial due to recombination, which allows transfer of DNA between distant strains [2]. While recombination may maintain the genetic coherence of a species [3–5], theory suggests selection is necessary for diversification [6]. Bacterial populations generally comprise genetically and ecologically differentiated clusters [7–9], and several explanations have been offered for this [10–12]. For example, in the Ecotype Model [10], niche -specific adaptive mutations cause genome-wide selective sweeps that remove variability between isolates in the same the niche, resulting in genetically differentiated clusters in different niches. Recently, a model of ecological differentiation among sympatric recombining bacteria has been developed [13, 14]. In this model the differentiation is triggered by an acquisition of a few habitat-specific alleles through horizontal gene transfer. If recombination between habitats is limited, the result is gradual diversification, eventually creating genomically and ecologically distinct clusters. Unlike in the Ecotype Model, which assumes genome-wide sweeps, here the sweeps occur only at the habitat-specific genes, but the overall genetic differentiation happens more slowly because recombination unlinks the habitat specific genes from the rest of the genome. The resulting pattern has a small number of short regions with strong habitat association, while the majority of the genome is relatively uncorrelated with habitat, a pattern observed between two clusters of closely related *Vibrio* bacteria [13].

**Fig 1:**
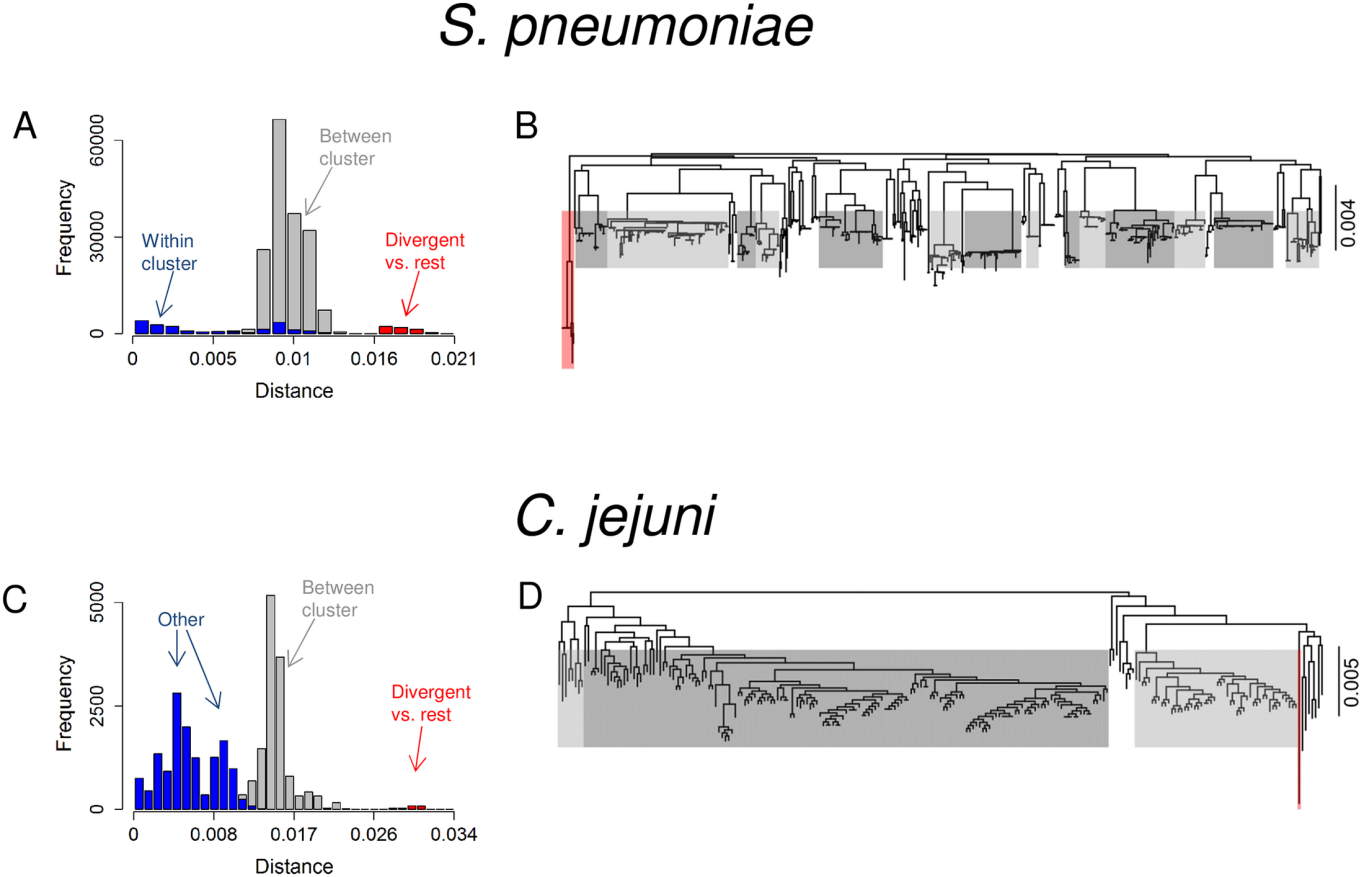
Population structures in *S. pneumoniae* and *C. jejuni* data sets. Distributions of pairwise distances computed between all strain pairs in the data sets (A,C), and the corresponding phylogenies (B,D). In the *S. pneumoniae* phylogeny (B), 16 previously identified sequence clusters are annotated as follows: the divergent cluster with red, 14 other monophyletic clusters with gray, and the remaining non-monophyletic cluster is not colored. Distances within and between these clusters are annotated in the distance histogram (A). Similarly, for *C. jejuni*, three clusters corresponding to separate branches of the phylogeny are colored with gray and one divergent strain with red (D), and the distances within and between these clusters are shown in the histogram (B). Annotation “Other” refers to within cluster comparisons as well as to distances between the non-colored strains and other strains.

Fig 1 shows population structures in data sets with 616 *Streptococcus pneumoniae* [15] and 235 *Campylobacter jejuni* samples [16–18] (see Materials and Methods). Both include strains divergent from the rest of the population, providing us with an opportunity to investigate the early stages of bacterial differentiation. In particular, the *S. pneumoniae* data consist of 16 sequence clusters (SCs) of which one, SC12, differs from the rest, and has previously been characterized as ‘atypical pneumococci’ representing a distinct species [15, 19]. All other SCs are at the same equilibrium distance from each other, maintained by recombination, corresponding to the main mode in the distance distribution [4]. Two additional modes can be discerned: one close to the origin comprising the within SC distances, which may be explained by selection of some sort [4], and the other representing the broad division of the data into SC12 vs. rest, which indicates less frequent recombination between these two clusters. Whether SC12 is a nascent cluster, which will continue to diverge, is not known. It is also possible that the distance could be an equilibrium produced by the combination of mutational divergence and occasional recombination with the parent cluster. A similar minor mode is found in *C. jejuni*, in this case arising from a single divergent isolate shown in red. Whether this is an isolate from a cluster in the early stages of divergence is similarly unknown.

**Fig 2:**
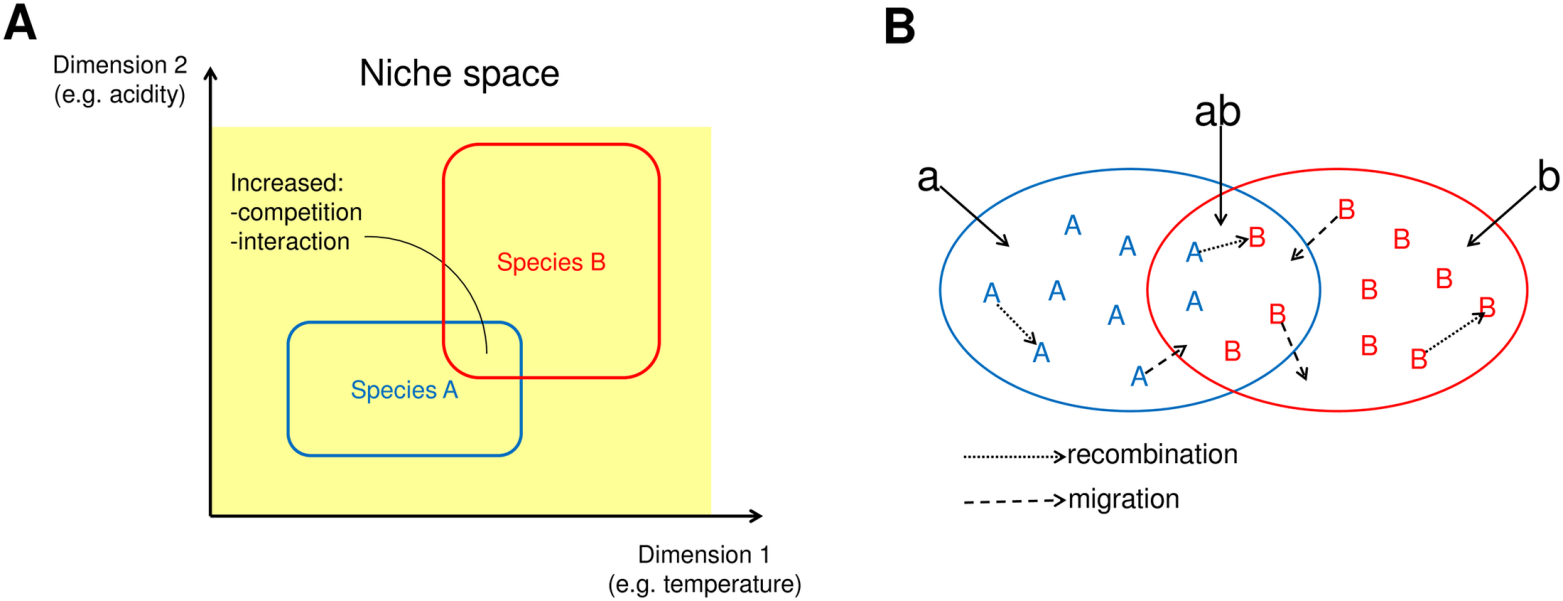
Motivation and the outline of the Overlapping Habitats Model. We model a situation where two species have overlapping ecological niches, and we assume increased competition and interaction inside the shared part (A). The Overlapping Habitats Model, outlined in B, assumes two types of strains, *A* and *B*, that live in habitats *a* or *b*, respectively. In addition, both types can live in the intersection of the habitats, denoted as *ab*. Type *A* strains can migrate between *a* and *ab* and type *B* strains between *b* and *ab*. Strain can only recombine with other strains in the same region of habitat space.

The goal to understand the population sub-divisions observed in Fig 1 motivated us to develop a model that could reproduce similar patterns. Previously models have been used to investigate the impact of homologous recombination on population structure [3, 20], the distribution of accessory genome [21–23], parallel evolution of the core and accessory genomes [4], migration and horizontal gene transfer [24], and gene sweeps and frequency dependent selection [25]. Our model is motivated by the fact that different species carry genetic differences that lead to physiological differences, and, consequently, to niche separation. However, the niche separation between different species may be incomplete, which means partial competition of the same resources and increased opportunities for interaction, as illustrated in Fig 2A. We take the model of sympatric differentiation [13, 14] as our starting point, and extend it in two ways. First, we introduce an explicit, controllable barrier for recombination between the two populations, and second, we derive an analytical approximation for the model.

An outline of our ‘Overlapping Habitats Model’ is shown in Fig 2B. Here the habitats represent different niches, and the key characteristic is the existence of two populations of different types of strains living in partially overlapping habitats. Recombination between the populations only occurs between individuals in the shared habitat, while migration enables strains to move between different parts of the habitat space. Notably, selection is implicit in the niche structure, in that there are regions of ecological space ‘private’ to each species where the other cannot survive. This habitat-specificity is assumed non-mutable and heritable, and could in practice be caused by a small number of genes. However, unlike [14], we do not model these explicitly, but rather focus on the consequences of that adaptation for the differentiation at the rest of the genome. This formulation facilitates predictions for the evolution of the population structure, given certain amount of habitat overlap, and, on the other hand, learning parameter values that result in a given population structure as an equilibrium.

## Materials and methods

### Simulation model

As the basis of our model, we use a Wright-Fisher forward simulation of discrete generations, where each generation is sampled with replacement from strains in the previous generation. In our model, a strain is represented by a collection of genes, similar to [2], and we assume the genes are ‘core’, i.e., present in all strains. Genes are encoded as binary sequences of fixed length (500 bp). The model has in total four free parameters: mutation rate, homologous recombination rate, the proportion of habitat overlap, and migration rate. Mutations and recombinations take place between sampling of the generations. Mutations change one base in the target sequence, while recombination results in the whole gene of the recipient to be replaced by the corresponding gene of the donor. Recombination is allowed only between strains within the same habitat, and accepted with probability that declines with respect to increasing sequence divergence [26–28]. The habitat overlap parameter specifies the size of the shared habitat, and migration determines the rate with which strains move between the shared and private habitats (see below). In contrast with [2, 4], we simulate complete binary sequences, avoiding the need for additional approximations.

In detail, we simulate a population of strains of two types, *A* and *B*, that live in habitats *a* and *b*, respectively; however, part of the habitat space, denoted by *ab*, is shared, and both strain types can inhabit it. For simplicity, the habitat-specificity encoding genes are assumed implicit and not simulated in the model, and we further assume that strain types can not be changed by recombination or mutation. Migration of type *A* strains between habitats *a* and *ab* is achieved by sampling the next generation of strains in *a*, for example, from all type *A* strains such that strains in *ab* are sampled with a relative weight determined by the migration parameter. This corresponds to the assumption that strains within each habitat compete against each other and those trying to enter the habitat. In detail, the sampling scheme can be described as follows. We denote by *A*_*a*_ and *A*_*ab*_ type *A* strains that are currently in *a* or *ab* environments; *B*_*b*_ and *B*_*ab*_ are defined correspondingly. We sample strains for *a* with replacement from *A*_*a*_ and *A*_*ab*_ such that the probability of sampling a strain *x* is equal to

$$\begin{array}{c} \mathrm{Pr}\left(x\right)=\frac{1}{|A_{a}\left|+m\right|A_{ab}|},\;\text{if}\;x\in A_{a}, \end{array}$$ (1)

and

$$\begin{array}{c} \mathrm{Pr}\left(x\right)=\frac{m}{|A_{a}\left|+m\right|A_{ab}|},\;\text{if}\;x\in A_{ab}, \end{array}$$ (2)

where 0 ≤ *m* ≤ 1 is the migration parameter. Value *m* = 0 corresponds to no migration, in which case Eqs 1 and 2 reduce to sampling the next generation for environment *a* from strains already in that environment. On the other hand, *m* = 1 corresponds to unlimited migration, and the next generation is sampled with equal probability from all type *A* strains in both environments *a* and *ab*. Strains for the *b* environment are sampled similarly from strains in *b* and *ab* environments. Finally, strains for the *ab* environment are sampled according to

$$\begin{array}{cc} \mathrm{Pr}(x) & =\frac{1}{m|A_{a}\left|+m\right|B_{b}\left|+\right|A_{ab}\left|+\right|B_{ab}|},\\ & \text{if}\;x\in A_{ab}\;\text{or}\;x\in B_{ab}, \end{array}$$ (3)

and

$$\begin{array}{cc} \mathrm{Pr}(x) & =\frac{m}{m|A_{a}\left|+m\right|B_{b}\left|+\right|A_{ab}\left|+\right|B_{ab}|},\\ & \text{if}\;x\in A_{a}\;\text{or}\;x\in B_{b}. \end{array}$$ (4)

Thus, if *m* = 0, the next generation of strains for the *ab* environment is sampled from strains already in the environment. In the other extreme (*m* = 1), the strains are sampled from all strains in both populations.

R-code for running and fitting the model, both simulation and the deterministic approximation (see below), is available as S1 Code.

### Deterministic approximation of the model

We also derive a deterministic approximation of the Overlapping Habitats Model, which enables rapid prediction of the evolution of the population structure without simulating the actual sequences. The model is based on average distances between and within the different sub-groups of the whole population: *A*_*a*_, *A*_*ab*_, *B*_*ab*_, and *B*_*b*_ (see the previous sub-section). In detail, let **d** be a vector comprising all 4 within and 6 between distances possible for the four groups. In S1 Text, we derive a function *f* that expresses how the average distances in the next generation, d*, approximately depend on the distances **d** in the current generation:

$$\begin{array}{c} d^{*}=f\left(d\right). \end{array}$$ (5)

One of the main interests is to identify stationary points in the distance distribution, i.e., distances **d**, for which

$$\begin{array}{c} d=f(d) \end{array}$$ (6)

holds.

We have implemented two methods to solve Eq (6). The first consists of using the update rule Eq (5) repeatedly until **d** converges, in which case the stationarity condition Eq (6) is satisfied. The second way to solve Eq (6) is to use a quasi-Newton method, implemented in the *optim*-function of the R software, to minimize the objective function *h*, defined as follows:

$$\begin{array}{c} h\left(d\right)=||f\left(d\right)-{d||}_{2} \end{array}$$ (7)

$$={\left[\sum_{i=1}^{10}{\left(f_{i}-d_{i}\right)}^{2}\right]}^{\frac{1}{2}},$$ (8)

where *f*_*i*_ is the prediction for the *i*th element in the distance vector of the next generation, and *d*_*i*_ the current value of the corresponding element. In practice we have found useful a strategy of first running the Newton’s method, which is fast, followed by the robust sequential update procedure to confirm convergence.

### Model fitting

Our strategy for fitting the Overlapping Habitats Model to a particular data set can be summarized as follows: we first assume the population structure observed in the data set represents an equilibrium, and use the analytical approximation, together with estimated values from the literature when available, to learn the remaining parameters so that the result is the observed equilibrium. Hence, we assume the patterns seen in data are relatively stable, but we also compare to a model that assumes more rapid divergence, and present a way to distinguish between these two (see Results). After fitting the model using the deterministic approximation, we run the simulation, which takes the stochasticity into account, to determine how easy it is to escape the equilibrium.

**Fig 3:**
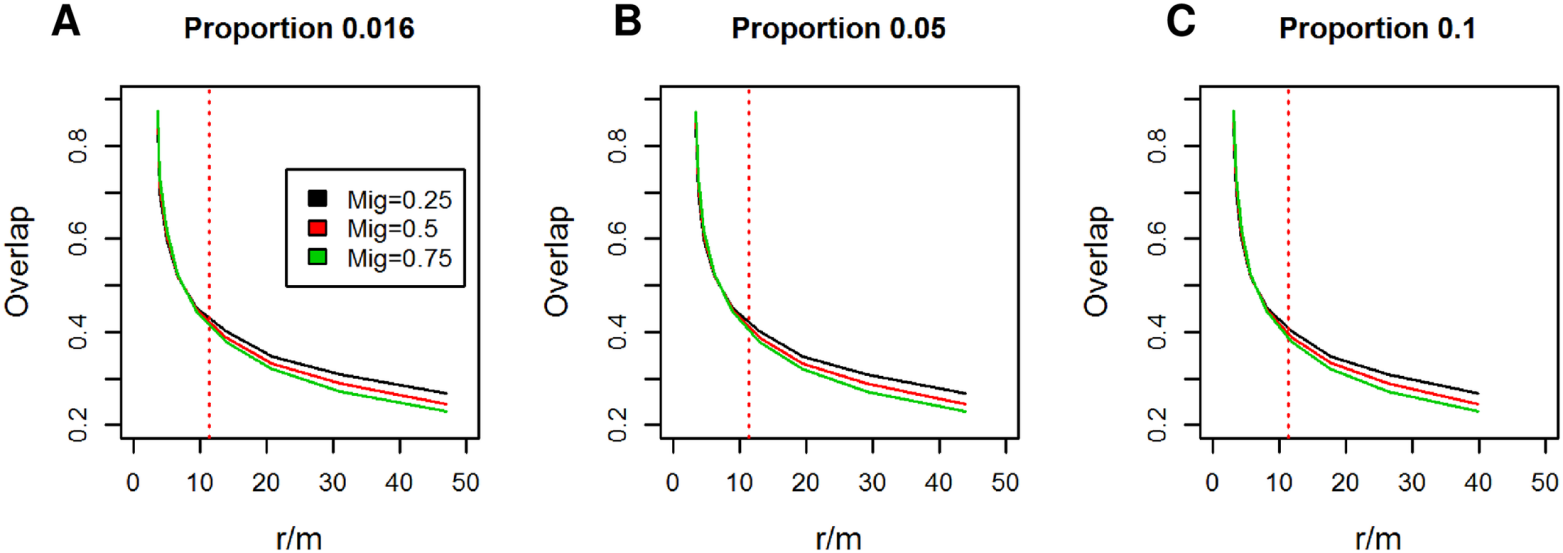
Fitting the model to the *S. pneumoniae* data. The panels show parameter combinations that produce the observed distance in the data between SC12 and the rest of the population as a stationary condition in the *S. pneumoniae* data. The *proportion* specifies the proportion of the divergent sub-population of the whole population (1.6% in the data), and panels A-C show results for different values of this parameter. It can be seen that several parameter combinations produce the same distance distribution. A previously reported value of *r*/*m* (=11.3) is marked with the vertical dotted line, and it determines the amount of overlap (∼41%). The results seem insensitive to both the proportion of strains in the divergent cluster and the migration rate, and we used values *proportion* = 0.05 and *migration* = 0.5.

As discussed above, the *S. pneumoniae* data can be broadly divided into two sub-populations. To estimate the habitat overlap, we assumed the population structure, i.e., the within and between sub-population distances observed, represented an equilibrium, with values *within* = 0.01, *between* = 0.017. Multiple parameter combinations produced these distances (Fig 3). Therefore, to determine the remaining parameters, we set the recombination rate, *r*/*m* to a previously reported value *r*/*m* = 11.3 [15]. The proportion of diverging strains of the whole population was set to 5%, and migration to 0.5 (results were insensitive to these choices, see Fig 3 and Results). These specifications led to an estimate of 41% habitat overlap, and a mutation rate of 2.4 mutations per generation per gene in the whole population.

The parameters for the *C. jejuni* were estimated similarly. In detail, we assumed that the *within* population distance was 0.015 (the main mode) and the *between* distance 0.03 (the small separate mode). We fixed the recombination rate to a plug-in estimate of *r*/*m* = 49, derived from an estimate that 98 percent of substitutions in MLST genes in the species are due to recombinations [29]. We again set the proportion of the diverging strains to be 5% of the whole population. These specifications yielded an estimate of 24% habitat overlap, and a mutation rate of 3.8 mutations per generation per gene in the whole population.

For both data sets, we set the total number of strains simulated as 10,000 and the number of genes as 30. As each gene had length 500, this corresponded to the total genome size of 15,000 bp. The probability of accepting a recombination was assumed to decline log-linearly with respect to the distance between the alleles in the donor and recipient strains, according to 10^−*Ax*^, where *x* is the Hamming distance between the alleles. We used *A* = 18 for the parameter that determines the rate of the decline, according to empirical data [2]. Before computing the ecoSNP summaries (see below) we sampled subsets of simulated strains whose sizes matched the sizes of the clusters in the data sets.

### Data sets

Core gene alignments and the cluster annotation of the *S. pneumoniae* strains were obtained from [15]. As an additional data cleaning step, we removed all genes with alignment lengths less than 265bp, which corresponded to the 0.05th quantile of the lengths of the alignments of the core genes. This step was added to increase confidence in the genes detected. This left us with 1,191 core genes in the 616 pneumococcal isolates. More specifically, the genes are here clusters of orthologous groups (COGs), and we use these terms interchangeably.

The *C. jejuni* data consisted of 239 previously published genomes [16–18]. From the reference-based assemblies mapped to the NCTC11168 reference genome, we extracted 423 COGs using ROARY [30] with default settings. As a data cleaning step, we removed four isolates with significantly increased levels of missing data. Additionally, we removed COGs with alignment lengths less than the 0.05th quantile (225bp) of all lengths. This left us with 401 COGs in 235 isolates. The divergent isolate in Fig 1 differs from others in terms of its sampling location (New Zealand), and by being the only isolate sampled from ‘environment’ and having ST = 2381.

## Results

### Overlapping Habitats Model predicts varying rates of divergence

**Fig 4:**
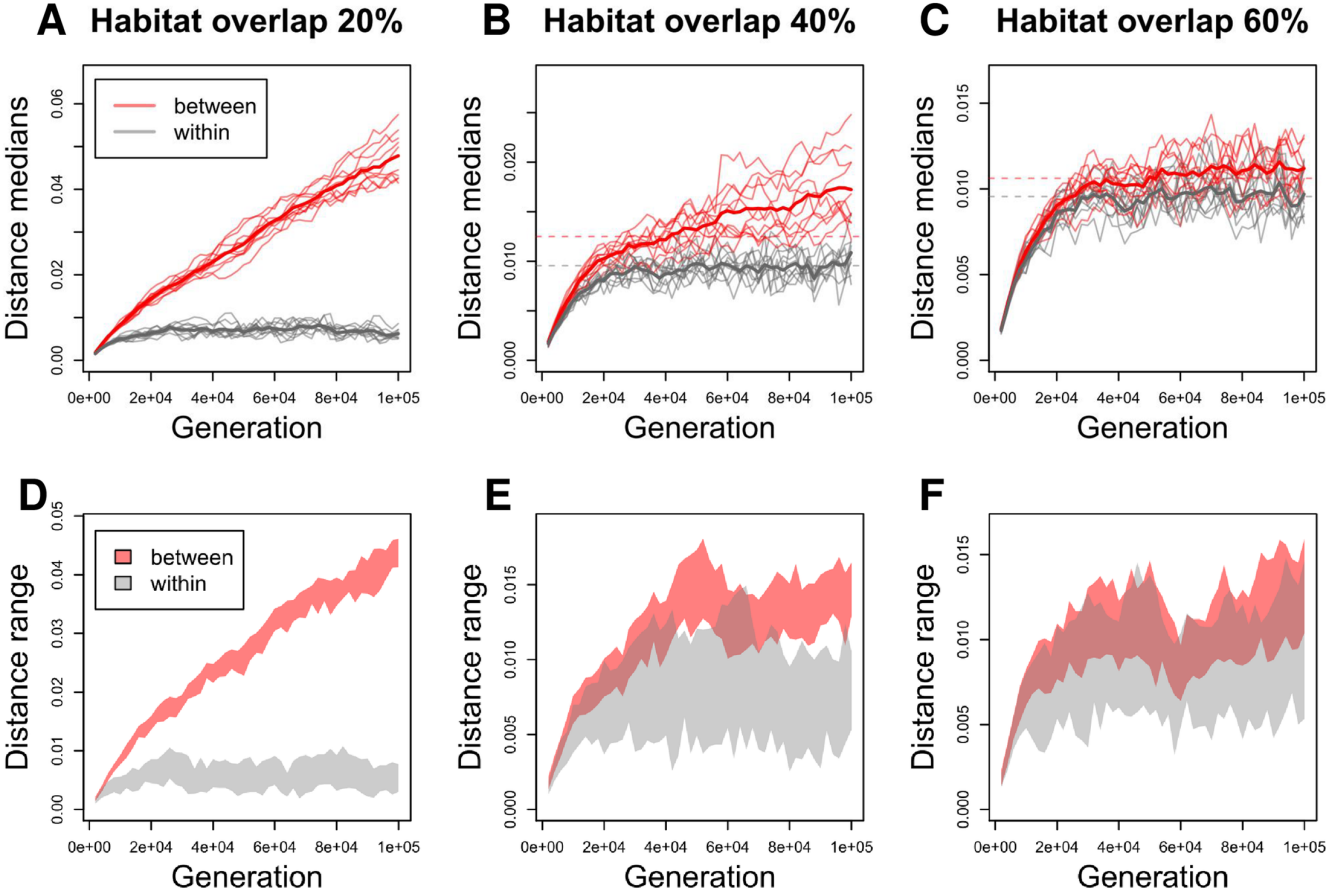
Simulation results from the Overlapping Habitats Model. A-C: the evolution of distances within and between strain types in simulations with 10^5^ generations. The solid thin red and gray lines show the median between and within strain type distances in ten repetitions, and the thick lines show the averages across the repetitions. The dashed horizontal lines in B,C show the predicted equilibrium distances from the deterministic approximation; in A the deterministic model did not have a solution. D-F show distance intervals between 0.1th and 0.9th quantiles in one randomly selected simulation (two additional simulations are shown in S1 Fig).

To investigate the impact of habitat structure on population structure, we simulated the model for 100,000 generations with two clusters, each with 5,000 strains. We varied the habitat overlap and migration, but used realistic mutation and recombination rates corresponding to the *S. pneumoniae* (see Materials and Methods). Fig 4 shows the evolution of the within and between cluster distances during the simulation. With the smallest overlap (Fig 4A and 4D), the limited interaction resulted in rapid divergence of the clusters, although within cluster distances reached an equilibrium as expected [2, 4]. With the largest overlap (Fig 4C and 4F) two clusters emerged, with the between cluster distance exceeding the within distance. However the clusters did not proceed to full separation, but rather maintained an equilibrium level of separation, and, furthermore, the between distances overlapped with the within distances, making clusters difficult to distinguish (Fig 4F). With an intermediate overlap (Fig 4B and 4E) the simulation still had periods of stationary behavior; however, now the clusters slowly drifted apart as a result of genes one by one escaping the equilibrium. To understand the equilibrium, we first note that if two clusters are very close, then recombination between them does not make them any more similar. If the clusters are very distant, the ability to recombine vanishes. The equilibrium, if exists, is located at an intermediate distance where the cohesive force of recombination equals the diversifying force of mutation.

### Accuracy of the deterministic approximation

Investigation of Fig 4 reveals that the deterministic approximation predicts the simulated within cluster distances with high accuracy. Also, with the smallest overlap, the deterministic approximation does not have a solution, immediately predicting the rapid divergence. However, we also see that the approximation has a tendency to underestimate the between cluster distances. The reason for this is that the deterministic approximation is based on average distances, and therefore does not account for variation in distances between specific donor and recipient alleles, whereas in the simulation distant recombinations, which have the biggest impact, are accepted less often. Therefore the approximation slightly overestimates the impact of recombination. Also, because the approximation is non-stochastic, it can not determine how easy it is to escape the equilibrium. Therefore, in our analyses of genomic data sets (see below), we first estimated the parameters with the deterministic approximation, and then ran the simulation with the learned values to produce the final prediction. S2–S4 Figs show additional results about the impacts of migration and recombination rates, and unequal cluster sizes, with similar conclusions. One interesting finding is that as long as migration is not extremely small (<0.01), its value has a negligible impact on the population structure (S2 Fig), motivating the use of a fixed value (*migration* = 0.5) in analyses of genomic data sets.

### Divergence rates in *S. pneumoniae* and *C. jejuni*

We next investigated whether the population divisions in the *S. pneumoniae* and *C. jejuni* data (Fig 1) are best explained by rapid clonal divergence, a stationary equilibrium, or some intermediate of these. To fit the Overlapping Habitats Model, representing the equilibrium or slow divergence, we assumed the distances between the divergent strains and other strains to be at equilibrium, and used a plug-in recombination rate estimate from the literature to compute the approximate overlap that would produce the observed level of separation (see Materials and Methods). For both data sets, a simulation with these parameters resulted in two separate clusters that were diverging slowly, with rates of 0.32 (*S. pneumoniae*) and 0.45 (*C. jejuni*) relative to the clonal divergence rates. This indicates the separation between the clusters, especially in the *C. jejuni* which also has a higher clonal divergence rate (see Model fitting), has exceeded the level where recombination could prevent the divergence. However, these results alone do not yet allow us to separate the two possible explanations: first, the clusters are in the process of slow divergence, as just described, or second, the clusters are in the process of rapid clonal diversification, and the distance between them just happens momentarily to be as observed.

### EcoSnp distribution separates fast and slow divergence

**Fig 5:**
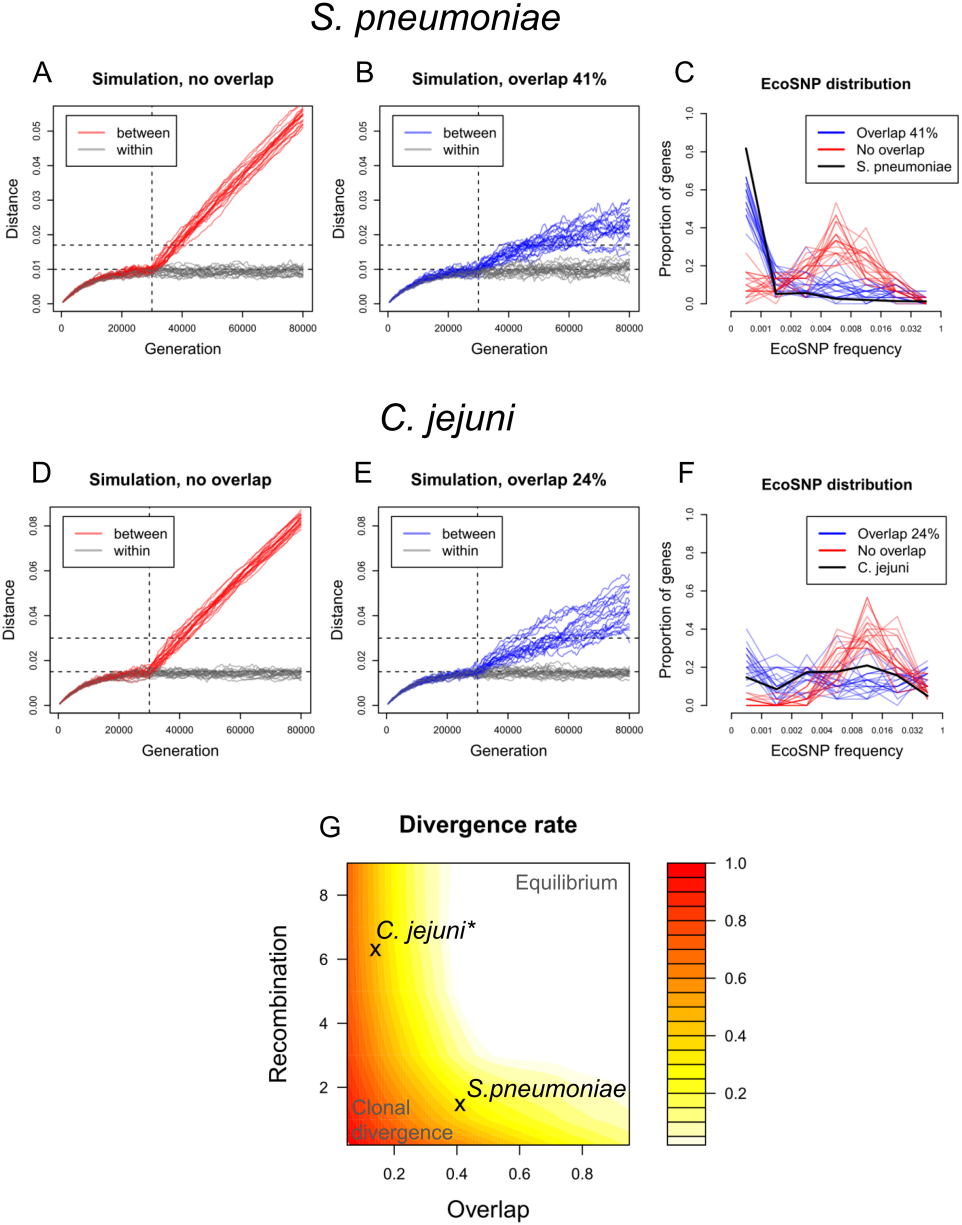
Comparing model output with the *S. pneumoniae* and *C. jejuni* data, and a summary of divergence rates. For each data set, we simulated the Overlapping Habitats Model 20 times without overlap (A,D) and with the estimated overlap (B,E). A barrier representing the size of the overlap between the clusters was introduced at the 30,000th generation (dashed vertical line) after which the clusters diverged. The horizontal lines show for reference the within and between cluster distances in *S. pneumoniae* and *C. jejuni*. Simulated ecoSNP distributions with and without overlap, computed at the generation when the simulated between-cluster distance matched the observed value, are compared with the observed ecoSNP distributions (C,F). Panel G summarizes the simulated rate of divergence between the two clusters. Color scale shows the rate relative to clonal divergence, averaged over the second half of the simulation. (*the heatmap is based on the mutation rate in *S. pneumoniae*, and, therefore, the location of *C. jejuni* is modified by moving it to the closest contour line corresponding to the divergence rate estimated using its own mutation rate, for which results are shown in S7 Fig)

A detailed comparison of the models’ outputs revealed a systematic difference in the ecoSNP distributions between the scenarios of clonal divergence vs. equilibrium or slow divergence, where ecoSNPs are defined, as in [13], as variants present in all strains of one cluster and absent from all strains of the other cluster. In particular, with rapid divergence and little recombination between the clusters, the ecoSNPs started to accumulate in all genes soon after the introduction of the recombination barrier (S5 and S6 Figs). On the other hand, under the equilibrium the majority of ecoSNPs were concentrated in only a few genes that already had escaped the equilibrium, while the majority of genes had no ecoSNPs at all during the whole simulation. For both data sets, the ecoSNP distribution supports the interpretation that the observed population structure is a result of equilibrium or slow divergence, rather than rapid clonal divergence (Fig 5). In the *S. pneumoniae* data the observed proportion of genes with no ecoSNPs is even higher than predicted by the overlap model, suggesting that previously published recombination rates may be underestimates. We note that while quantitatively the simulation output depended on the exact parameter values, qualitatively the conclusions regarding the main patterns were robust across a wide range of parameter values.

## Discussion

Here we have shown that certain combinations of niche structure and recombination may result in stable but distinct clusters, creating what might be termed ‘satellite species’, as seen in *S. pneumoniae*, and that these may be distinguished from dynamically diverging clusters using ecoSNPs, as shown by the analysis of *C. jejuni*. Having shown stable clusters are possible in nature, future work will be able to focus on determining the exact dimensions of the niches and candidate loci associated with them. We should also note that ‘niche’ is here an abstraction, similar to that proposed by Hutchinson [31], as the hypervolume in resource space where a species can survive, which we consider a proxy for physical connectedness. However, we extend this to be the portion of resource space where recombination is possible. In some cases recombination might occur without direct contact between the organisms, such as if mediated by diffusing DNA, and in this case the two will not be exactly equivalent. These simplifying assumptions are intended to help make a simple model, applicable to multiple species, that can be developed further in future work.

There are several differences between our model and previous work. Notably, selection is implicit in the niche structure, in that there are regions of ecological space ‘private’ to each species where the other cannot survive. This distinguishes the niches in question from purely geographic separation. The strict fitness threshold was selected for simplicity, and could be extended to a more realistic situation where strains have some probability of surviving in different niches, at the cost of introducing additional parameters to the model. We have chosen this approach as a way of implicitly modeling selection on already ecologically differentiated clusters (or species), because our interest is in the consequences of this ecological differentiation for parts of the genome that are not directly involved in niche specificity and are able to recombine. Rather than assuming niche specifying genes themselves cannot be recombined in reality, we suspect our model approximates the case where niche specificity is due to multiple loci, such that transfer of one (or a few) is not sufficient to alter a strain’s niche or a cluster’s trajectory.

Key parameters in our model are mutation rate, recombination rate, proportion of habitat overlap, and migration rate. Within-population distances were found informative about mutation rates, and values from the literature were available for recombination rates. To understand how the habitat overlap can be learned, we first note that if recombination between clusters happens freely at the same rate as within clusters, a certain equilibrium distance between the clusters is predicted. Observed distance greater than this suggests some additional barrier for recombination, and the extent of the barrier can be learned to produce that distance. The habitat structure can be interpreted as this additional barrier. In detail, the reduction in recombination between populations equals 1 − *p*, where *p* is the proportion of pairs that can recombine of all pairs (i.e. *p* = (|*A*_*ab*_||*B*_*ab*_|)/(|*A*||*B*|)). Notably migration does not affect the amount of genetic exchange between the populations, but only homogenizes each internally. Consequently, any non-negligible migration rate produced similar results. This finding motivated the simplification of our model by fixing the migration parameter. Eventually, after fitting the model, the ecoSNP distribution can be used to determine whether the fitted model, representing equilibrium or slow divergence, is better suited to explain the population structure than a model of more rapid clonal divergence.

The concentration of ecoSNPs in a few genome regions has previously been taken as evidence for gene-specific sweeps of habitat-specific adaptive alleles acquired through horizontal gene transfer [13]. Our results suggest a similar pattern may emerge without explicit selection on the loci affected, as a result of reduction in recombination due to habitat structure, which may allow a region to drift sufficiently far apart to reduce the ability for genetic exchange in the locus even further. This is followed by rapid diversification within the region concerned, while the rest of the genome remains at equilibrium. This recalls the concept of ‘fragmented’ speciation in which different parts of the genome speciate at different times [32], except here this was achieved without explicit selection on the diverging region. Eventually this results in highly divergent habitat-specific loci surrounded by regions with little habitat association. In practice this process could happen together with selection at the habitat-specific loci, as both processes have the potential to increase differentiation and create ecoSNPs between the clusters.

Despite its simplicity, the model adequately captured the main sub-divisions in two data sets. Nonetheless, much structure is not captured, for example the individual sequence clusters in the *S. pneumoniae* data. Our model does not contradict this additional structure, but instead shows that the individual sequence clusters can indeed be ecologically different, and still maintain the equilibrium distance between them, as a mere 60% of habitat overlap is sufficient for this (Fig 4). Nevertheless, the dense clusters observed in the data likely require some additional form of selection. While some alternatives are discussed in [4], we expect that in practice the within species dynamics will be governed by far more niches, with subtle distinctions leading to far more overlap, and we are actively working to extend the present work to handle this and see if it can at least qualitatively produce substructure like that we see in the pneumococcus. To conclude, our model provides means to characterize equilibrium structures and define speciation trajectories in bacterial populations and we believe it will be helpful when interpreting similar patterns in other data sets.

## Supporting information

S1 Text Derivation of the deterministic approximation for the Overlapping Habitats Model. (PDF)

S1 Fig Two additional distance range examples. The figure shows distance range results, interpreted in exactly the same way as Fig 4D–4F in the main text. Rows represent independent simulations of the model, and columns different amounts of habitat overlap.
(PDF)

S2 Fig Impact of migration in the simulation. Each panel shows median within and between distances in 10 independent simulation runs. Columns represent different amounts of habitat overlap, and rows different migration rates. We see that the results are almost identical for migration≥0.01, and even with migration = 0.001, the results are still qualitatively similar.
(PDF)

S3 Fig Impact of unequeal population sizes in the simulation. The simulation results in Fig 4 in the main text were based on simulation of 5,000 strains of both types. Here we repeat this with exactly the same parameters, except that 2,000 type *A* strains and 8,000 type *B* strains were simulated. The first row shows the within distances in the smaller and the second row in the larger population. The same between distances are shown on both rows. We see that in the larger population there is more diversity than in the smaller one. Nevertheless, the deterministic approximation accurately predicts the within distances in both populations.
(PDF)

S4 Fig Impact of recombination rate in the simulation. The top row shows results with 1/3 and the bottom row 3 times the recombination rate compared to that in Fig 4 in the main text. We see that the between population distance decreases when recombination rate is increased. As has been explained before, the within population equilibrium distance is not affected by the recombination rate, as long as recombination is high enough for the equilibrium to emerge [2, 4].
(PDF)

S5 Fig Evolution of ecoSNP distribution in the *S. pneumoniae* simulation. The solid curve shows the median of the ecoSNP distribution, the dashed curves the 0.1th and 0.9th quantiles. The top row corresponds to the simulation from the Overlapping Habitats Model, fitted to the *S. pneumoniae* data, and the bottom row the corresponding clonal simulation. The colums show results for three independent simulations. The vertical line marks the generation when the between distance matched that observed in the *S. pneumoniae* data. We see that in the Overlapping Habitats Model (top row) the majority of genes had very few ecoSNPs throughout the simulation, although some genes started to accumulate ecoSNPs immediately after the barrier between the populations had been introduced. In clonal divergence all genes accumulated ecoSNPs at an approximately constant rate.
(PDF)

S6 Fig Evolution of ecoSNP distribution in the *C. jejuni* simulation. The results are interpreted in the same way as those in S5 Fig.
(PDF)

S7 Fig Simulated divergence rates using mutation rate estimated for *C. jejuni*. The ‘x’ shows the predicted rate for *C. jejuni*.
(PDF)

S1 Code R-code to run the model. (ZIP)
