# Supplementary material for: Speciation trajectories in recombining bacterial species
Source: PLoS Comput Biol. 2017 Jul 3;13(7):e1005640. doi: 10.1371/journal.pcbi.1005640 (PMC5542674; doi:10.1371/journal.pcbi.1005640)
Supplement: S1 Text — (PDF) [file pcbi.1005640.s001.pdf]

Speciation trajectories in recombining bacterial species - Supplementary Text S1

Pekka Marttinen, William P. Hanage

Contents

1 Derivation of the deterministic approximation for distance evolution 1
1.1 Notation 1
1.2 Update equations for $d(A_a)$ 2
1.3 Update equations for $d(A_a, A_{ab})$ 3
1.4 Update equations for $d(A_{ab})$ 3
1.5 Update equations for $d(A_a, B_{ab})$ 4
1.6 Update equations for $d(A_{ab}, B_{ab})$ 4
1.7 Update equations for $d(A_a, B_{ab})$ 4
1.8 Detailed derivations 5
1.8.1 Lemma 1 5
1.8.2 Impact of mutation on $d(A_a)$ 6
1.8.3 Impact of recombination on $d(A_a)$ 6
1.8.4 Impact of sampling on $d(A_a)$ 6
1.8.5 Impact of recombination on $d(A_a, A_{ab})$ 7

1 Derivation of the deterministic approximation for distance evolution

1.1 Notation

Here we describe how to compute the distances between different parts of the population in the next generation approximately, if the distances in the current generation are known. The parts correspond to groups of strains with the same environment and type. To introduce some notation, let X_y denotes strains of type X in environment y. With this notation, the population can be divided into four parts: A_a, A_{ab}, B_{ab}, B_b, where, for example, A_a is the set of A strains in environment a. Furthermore, let $S^z_{x,y}$ denote the set of strains of type z that are sampled from environment x to be part of the next generation in environment y. For example, $S^A_{a,ab}$ denotes type A strains that are sampled from environment a and are part of the next generation of strains in environment ab. Fig. 1 summarizes the notation.

Distance function is denoted by $d()$ and, depending on the arguments provided, gives the within or between group distance or the distance between individual strains. The average distance between strains within a group X is denoted by $d(X)$. For example, $d(A_{ab})$ denotes the average distance between A strains in environment ab. Furthermore, $d(X, Y)$ denotes the average distance between strains in groups X and Y. Individual strains are denoted by lower-case letters, such as x or y, and their distance is denoted by $d(x, y)$. The distances are represented as the number of differing sites between the strains.

Next we describe approximately how the distances between different parts of the whole population evolve. In total, there are 10 different unknown distances, out of which four are within distances: $d(A_a)$, $d(A_{ab})$, $d(B_{ab})$, $d(B_b)$ and six between distances: $d(A_a, A_{ab})$, $d(A_a, B_{ab})$, $d(A_a, B_b)$, $d(A_{ab}, B_{ab})$, $d(A_{ab}, B_b)$, $d(B_{ab}, B_b)$. Due to symmetry reasons - for example $d(A_a)$ and $d(B_b)$ evolve similarly after switching both the environment and the strain type - it is sufficient to show the update equations for six distances: $d(A_a), d(A_{ab}), d(A_a, A_{ab}), d(A_a, B_{ab}), d(A_a, B_b), d(A_{ab}, B_{ab})$. Each of the distances is affected in different ways by sampling, mutation and recombination. Mutation generally increases distances, while

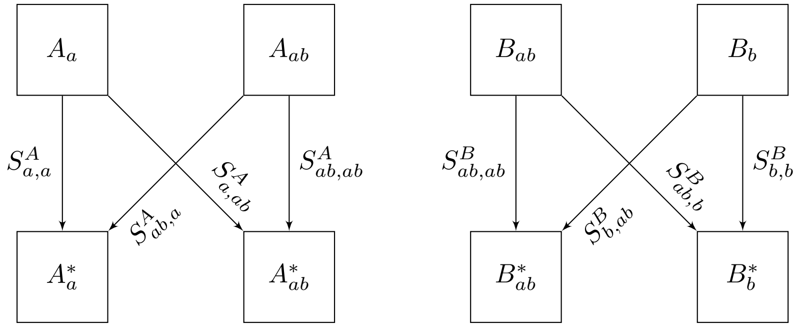

Figure 1: Notation used in the derivations. The upper row represents the four parts of the population, corresponding to strain types in different environments in the current generation. The lower row represents the next generation after sampling. The arrows indicate the directions of the strains sampled.

recombination and sampling may either increase or decrease distances, depending on the parameters of the model and the current distances. For example, if migration $m = 0$, then $d(A_a)$ decreases a little due to sampling, because all strains for the next generation are sampled from the strains in A_a, but with an increased number of identical strains. On the other hand, if migration is high, $d(A_a)$ may increase due to the fact that strains from a different part of the population, A_{ab}, are entering A_a. As another example, $d(A_{ab}, B_{ab})$ decreases due to recombinations, because A_{ab} and B_{ab} can donate alleles to each other. A by-product of this is that $d(A_a, A_{ab})$ tends to increase, when alleles from B_{ab} enter A_{ab}, making A_{ab} at the same time more distant from A_a.

Below we provide approximate formulas for the impact of mutation, recombination, and sampling, on each of the six distances. Finally, we provide detailed derivations for a representative set of the formulas, demonstrating techniques which were used to derive all the other formulas also. We denote by $d^*()$ the distances after applying a specific operation on the original distances $d()$. Each of the update equations was validated by comparing the predicted change with multiple simulations of the corresponding step of the algorithm (sampling, adding mutations/recombinations). With the exception of the impact of recombination, which was found to be slightly overestimated by the formulas (see Section 1.8.3 for a detailed discussion), all other updates accurately predicted the expected outcome of the simulation.

Additional notation used: G: the number of genes simulated for each strains; L: the length of each gene in basepairs; $|\cdot|$ the cardinality operator, for example $|A_a|$ is the number of strains in A_a; θ : mutation rate (the probability of a mutation occurring at a certain site in a certain genome in one generation); R: recombination rate (the expected number of recombination attempts in one gene in the whole population per one generation); N the total number of strains, *i.e.*, $N = |A_a| + |A_{ab}| + |B_{ab}| + |B_b|$.

1.2 Update equations for $d(A_a)$

The formulas provided in this section show how to compute the approximate distance within the A_a group of strains in the next generation, given parameters and the current distances within and between the diffent groups. In particular, the distance is affected by mutation, recombination, and sampling (which involves the impact of migration), and the total effect on the resulting distance is obtained as a sum of these three components.

Impact of mutation

$$d^*(A_a) = d(A_a) + 2LG\theta$$

(*This result will be derived in detail in Section 1.8.2.*)

Impact of recombination

$$d^*(A_a) = d(A_a)$$

(*This result will be derived in detail in Section.1.8.3*)

Impact of sampling

$$d^*(A_a) = \binom{|A_a|}{2}^{-1} \left[\binom{|S^A_{a,a}|}{2} d(A_a) \left(1 - \frac{1}{|A_a|}\right) + \ldots \right.$$
$$|S^A_{a,a}||S^A_{ab,a}| d(A_a, A_{ab}) + \ldots$$
$$\left. + \binom{|S^A_{ab,a}|}{2} d(A_{ab}) \left(1 - \frac{1}{|A_{ab}|}\right) \right]$$

(*This result will be derived in detail in Section 1.8.4.*)

1.3 Update equations for $d(A_a, A_{ab})$

Impact of mutation

$$d^*(A_a, A_{ab}) = d(A_a, A_{ab}) + 2LG\theta$$

Impact of recombination

$$d^*(A_a, A_{ab}) = d(A_a, A_{ab}) + \frac{|B_{ab}|R}{N(N-1)} 10^{-\frac{\xi}{GL} d(A_{ab}, B_{ab})} \left[d(A_a, B_{ab}) - d(A_a, A_{ab}) \right]$$

(*This result will be derived in detail in Section 1.8.5.*)

Impact of sampling

$$d^*(A_a, A_{ab}) = \frac{1}{|A_a||A_{ab}|} \left\{ |S^A_{a,a}||S^A_{ab,ab}| d(A_a, A_{ab}) + \ldots \right.$$
$$|S^A_{a,a}||S^A_{a,ab}| d(A_a) \times \left(1 - \frac{1}{|A_a|}\right) + \ldots$$
$$|S^A_{ab,a}||S^A_{ab,ab}| d(A_{ab}) \times \left(1 - \frac{1}{|A_{ab}|}\right) + \ldots$$
$$\left. |S^A_{a,ab}||S^A_{ab,a}| d(A_a, A_{ab}) \right\}$$

1.4 Update equations for $d(A_{ab})$

Impact of mutation

$$d^*(A_{ab}) = d(A_{ab}) + 2LG\theta$$

Impact of recombination

$$d^*(A_{ab}) = d(A_{ab}) + \frac{2|B_{ab}|R}{N(N-1)} 10^{-\frac{\xi}{GL} d(A_{ab}, B_{ab})} \left[d(A_{ab}, B_{ab}) - d(A_{ab}) \right]$$

Impact of sampling

$$d^*(A_{ab}) = \binom{|A_{ab}|}{2}^{-1} \left\{ \binom{|S^A_{ab,ab}|}{2} d(A_{ab}) \times \left(1 - \frac{1}{|A_{ab}|}\right) + \ldots \right.$$
$$|S^A_{a,ab}||S^A_{ab,ab}| d(A_a, A_{ab}) + \ldots$$
$$\left. \binom{S^A_{a,ab}}{2} d(A_a) \times \left(1 - \frac{1}{|A_a|}\right) \right\}$$

1.5 Update equations for $d(A_a, B_{ab})$

Impact of mutation

$$d^*(A_a, B_{ab}) = d(A_a, B_{ab}) + 2LG\theta$$

Impact of recombination

$$d^*(A_a, B_{ab}) = d(A_a, B_{ab}) + \frac{|A_{ab}|R}{N(N-1)} 10^{-\frac{\xi}{GL} d(A_{ab}, B_{ab})} \left[d(A_a, A_{ab}) - d(A_a, B_{ab}) \right]$$

Impact of sampling

$$\begin{aligned} d^*(A_a, B_{ab}) = \frac{1}{|A_a||B_{ab}|} \{ & |S^A_{a,a}||S^B_{ab,ab}| d(A_a, B_{ab}) + \ldots \\ & |S^A_{ab,a}||S^B_{ab,ab}| d(A_{ab}, B_{ab}) + |S^A_{a,a}||S^B_{b,ab}| d(A_a, B_b) + \ldots \\ & |S^A_{ab,a}||S^B_{b,ab}| d(A_{ab}, B_b) \} \end{aligned}$$

1.6 Update equations for $d(A_{ab}, B_{ab})$

Impact of mutation

$$d^*(A_{ab}, B_{ab}) = d(A_{ab}, B_{ab}) + 2LG\theta$$

Impact of recombination

$$\begin{aligned} d^*(A_{ab}, B_{ab}) = d(A_{ab}, B_{ab}) - \{ & |B_{ab}| \left[d(A_{ab}, B_{ab}) - d(B_{ab}) \right] + \ldots \\ & |A_{ab}| \left[d(A_{ab}, B_{ab}) - d(A_{ab}) \right] \} \times \frac{R}{N(N-1)} 10^{-\frac{\xi}{GL} d(A_{ab}, B_{ab})} \end{aligned}$$

Impact of sampling

$$\begin{aligned} d^*(A_{ab}, B_{ab}) = & \frac{1}{|A_{ab}||B_{ab}|} \{ |S^A_{ab,ab}||S^B_{ab,ab}| d(A_{ab}, B_{ab}) + \ldots \\ & |S^A_{a,ab}||S^B_{ab,ab}| d(A_a, B_{ab}) + |S^A_{ab,ab}||S^B_{b,ab}| d(A_{ab}, B_b) + \ldots \\ & |S^A_{a,ab}||S^B_{b,ab}| d(A_a, B_b) \} \end{aligned}$$

1.7 Update equations for $d(A_a, B_{ab})$

Impact of mutation

$$d^*(A_a, B_{ab}) = d(A_a, B_{ab}) + 2LG\theta$$

Impact of recombination

$$d^*(A_a, B_{ab}) = d(A_a, B_{ab}) + \frac{|A_{ab}|R}{N(N-1)} \times 10^{-\frac{\xi}{GL} d(A_{ab}, B_{ab})} \left[d(A_a, A_{ab}) - d(A_a, B_{ab}) \right]$$

Impact of sampling

$$\begin{aligned} d^*(A_a, B_{ab}) = \frac{1}{|A_a||B_{ab}|} \{ & |S^A_{a,a}||S^B_{ab,ab}| d(A_a, B_{ab}) + \ldots \\ & |S^A_{ab,a}||S^B_{ab,ab}| d(A_{ab}, B_{ab}) + |S^A_{a,a}||S^B_{b,ab}| d(A_a, B_b) + \ldots \\ & |S^A_{ab,a}||S^B_{b,ab}| d(A_{ab}, B_b) \} \end{aligned}$$

1.8 Detailed derivations

Here we provide detailed derivations for a representative subset of formulas from the previous sections. The other equations were derived similarly.

1.8.1 Lemma 1

Suppose we sample strains S from group A. Then, the average distance between the sampled strains is given by

$$d(S) = d(A)\left(1 - \frac{1}{|A|}\right)$$

Proof: In total $|S|$ strains are sampled with replacement from A. When A is large, each strain in A will have approxmately $Binomial(n = |S|, p = 1/|A|)$ descendants, which we further approximate with a $Poisson(\lambda)$ distribution, where $\lambda = |S|/|A|$. Let $I_i(k)$ denote an indicator that strain x_i has exactly k descendants. The expected number of strains having k descendants is therefore

$$\begin{aligned} E\left[\sum_{i=1}^{N} I_i(k)\right] &= |A| \times E\left[I_1(k)\right] \\ &= |A| \times \Pr(I_i(k) = 1) \\ &= |A| \times Poisson(k; \lambda) \\ &= |A| \times \exp(-\lambda)\frac{\lambda^k}{k!}. \end{aligned} \tag{1}$$

After sampling, every group of desendants of the same parent consists of identical strains. Therefore, the reduction in the total between-strain distances caused by a group of k desecendants with the same parent, is given by

$$\binom{k}{2} d(A). \tag{2}$$

Therefore, the total reduction in distances due to sampling identical strains, is obtained by multiplying (1) with (2) and summing over groups of different sizes

$$\begin{aligned} &\sum_{k=2}^{\infty} |A| \exp(-\lambda)\frac{\lambda^k}{k!}\binom{k}{2} d(A) \\ &= \frac{|A|d(A)\lambda}{2} \sum_{k=2}^{\infty} \exp(-\lambda)\frac{\lambda^{k-1}}{(k-1)!}(k-1) \\ &= \frac{|A|d(A)\lambda}{2} \sum_{k=1}^{\infty} \exp(-\lambda)\frac{\lambda^{k}}{k!}k \\ &= \frac{|A|d(A)\lambda^2}{2} = \frac{d(A)|S|^2}{2|A|}. \end{aligned}$$

The last line follows dy noticing that the summation in the second last line was equal to the expectation of a $Poisson(\lambda)$ distribution. Thus, we can write the average distance between strains in S as follows:

$$\begin{aligned} d(S) &= \binom{|S|}{2}^{-1}\left[\binom{|S|}{2} d(A) - \frac{|A|d(A)\lambda^2}{2}\right] \\ &= d(A) - \frac{2}{|S|\left(|S|-1\right)}\frac{d(A)|S|^2}{2|A|} \\ &\approx d(A) - \frac{d(A)}{|A|} = d(A)\left(1 - \frac{1}{|A|}\right) \end{aligned}$$

1.8.2 Impact of mutation on $d(A_a)$

The total expected number of mutations among A_a strains is equal to $|A_a|LG\theta$. We assume that each mutation increases the distance of the mutated strain to every other strain by 1. This assumption is accurate when most mutations intruduce new polymorphisms, which is true when sequences are very similar (having, for example, 0.99 percent of sites identical). Therefore, the average distance between the strains after mutations is obtained from

$$\begin{aligned} d^*(A_a) &= \binom{|A_a|}{2}^{-1}\left[\binom{|A|}{2}d(A_a) + |A_a|LG\theta\,(|A_a|-1)\right] \\ &= d(A_a) + 2LG\theta \end{aligned}$$

1.8.3 Impact of recombination on $d(A_a)$

The total number of recombination attempts in the whole population in all genes in one generation is equal to RG. Out of those, the number of attempts within strains in A_a is equal to

$$\frac{\binom{|A_a|}{2}}{\binom{N}{2}}RG = \frac{|A_a|(|A_a|-1)}{N(N-1)}RG.$$

Each recombination attempt is accepted with probability $10^{-\xi d}$, where d is the distance between the donor and recipient alleles (relative to the length of the recombining region). On average, the normalized distance between the donor and recipient strains in A_a is equal to $d(A_a)/LG$, and we plug-in this value to compute the approximate number of accepted recombinations within A_a, which we denote by $R(A_a)$:

$$R(A_a) \equiv \frac{|A_a|(|A_a|-1)}{N(N-1)}RG \times 10^{-\xi\frac{d(A_a)}{LG}}. \tag{3}$$

Using the average distance to compute the overall proportion of accepted recombinations is likely to lead to an overestimation of the impact of recombination, because recombinations between distant alleles, causing the biggest changes, are in reality less likely to be accepted than recombinations between close-by alleles. However, when we experimented with a range of recombination rate values, the analytical results were reasonably similar to the simulations, which used the distance between alleles when computing the acceptance probability of each specific recombination event.

By making the donor and recipient alleles equal, a single recombination decreases the total sum of within A_a distances by $d(A)/G$. Thus,

$$\begin{aligned} d^*(A_a) &= \binom{|A_a|}{2}^{-1}\left[\binom{|A_a|}{2}d(A_a) - R(A_a)\frac{d(A_a)}{G}\right] \\ &= \binom{|A_a|}{2}^{-1}\left[\binom{|A_a|}{2}d(A_a) - \frac{|A_a|(|A_a|-1)}{N(N-1)}R \times 10^{-\xi\frac{d(A_a)}{LG}} \times d(A_a)\right] \\ &= d(A_a)\left(1 - \frac{2R}{N(N-1)} \times 10^{-\xi\frac{d(A_a)}{LG}}\right) \\ &= d(A_a)(1 - O(1/N^2)) \\ &\approx d(A_a). \end{aligned}$$

1.8.4 Impact of sampling on $d(A_a)$

The next generation of strains in A_a consist of strains sampled from current A_a (denoted by $S^A_{a,a}$) and current A_{ab} ($S^A_{ab,a}$). Therefore, the updated within A_a is distance, $d^*(A_a)$, is a combination of current within A_a distances $d(A_a)$, current within A_{ab} distances $d(A_{ab})$, and current between A_a and A_{ab} distances $d(A_a, A_{ab})$. However, the reduction in distances between strains sampled from the same group, for example A_a, resulting from the fact that some strains are sampled multiple times, must be taken into

account, using Lemma 1.

$$\begin{aligned}
d^*(A_a) &= \binom{|A_a|}{2}^{-1} \left[\binom{|S^A_{a,a}|}{2} d(S^A_{a,a}) + |S^A_{a,a}||S^A_{ab,a}| d(A_a, A_{ab}) + \binom{|S^A_{ab,a}|}{2} d(S^A_{ab,a})\right] \\
&= \binom{|A_a|}{2}^{-1} \left[\binom{|S^A_{a,a}|}{2} d(A_a)\left(1 - \frac{1}{|A_a|}\right) + |S^A_{a,a}||S^A_{ab,a}| d(A_a, A_{ab}) + \dots \right. \\
&\left. + \binom{|S^A_{ab,a}|}{2} d(A_{ab})\left(1 - \frac{1}{|A_{ab}|}\right)\right]
\end{aligned}$$

1.8.5 Impact of recombination on $d(A_a, A_{ab})$

On average, a recombination from B_{ab} into A_{ab} changes the distance between the recipient A_{ab} strain and all A_a strains from $d(A_a, A_{ab})$ to $\frac{G-1}{G} d(A_a, A_{ab}) + \frac{1}{G} d(A_a, B_{ab})$. Thus, by denoting the number of recombinations from B_{ab} to A_{ab} by $R(B_{ab} \to A_{ab})$, the total impact of recombinations can be written as

$$\begin{aligned}
d^*(A_a, A_{ab}) &= d(A_a, A_{ab}) + \frac{1}{|A_a||A_{ab}|} R(B_{ab} \to A_{ab})|A_a| \left(\frac{G-1}{G} d(A_a, A_{ab}) + \frac{1}{G} d(A_a, B_{ab}) - d(A_a, A_{ab})\right) \\
&= d(A_a, A_{ab}) + \frac{R(B_{ab} \to A_{ab})}{|A_{ab}|G} \left[d(A_{ab}, B_{ab}) - d(A_a, A_{ab})\right]. \qquad (4)
\end{aligned}$$

We approximate the number of recombinations from B_{ab} into A_{ab} by

$$R(B_{ab} \to A_{ab}) \approx \frac{|A_{ab}||B_{ab}|}{N(N-1)} RG \times 10^{-\frac{\xi}{GL} d(A_{ab}, B_{ab})}, \qquad (5)$$

which follows from similar assumptions as those used when deriving equation (3). Subsituting (5) into (4) yields

$$d^*(A_a, A_{ab}) = d(A_a, A_{ab}) + \frac{|B_{ab}|R}{N(N-1)} \times 10^{-\frac{\xi}{GL} d(A_{ab}, B_{ab})} \left[d(A_a, B_{ab}) - d(A_a, A_{ab})\right].$$
