# Supplementary figures and images for: Speciation trajectories in recombining bacterial species
Source: PLoS Comput Biol. 2017 Jul 3;13(7):e1005640. doi: 10.1371/journal.pcbi.1005640 (PMC5542674; doi:10.1371/journal.pcbi.1005640)

**A** Habitat overlap 20%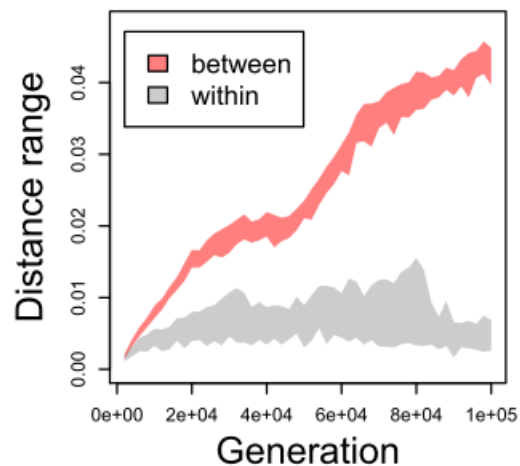**B** Habitat overlap 40%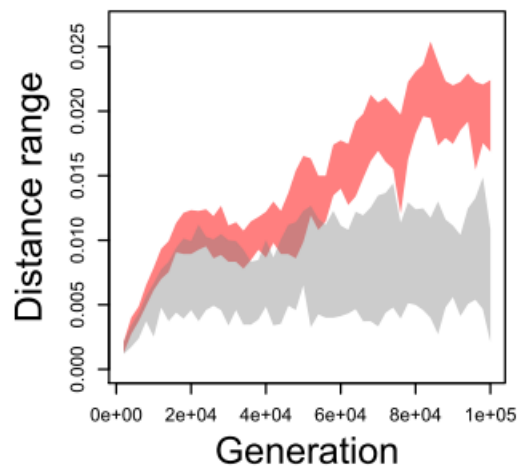**C** Habitat overlap 60%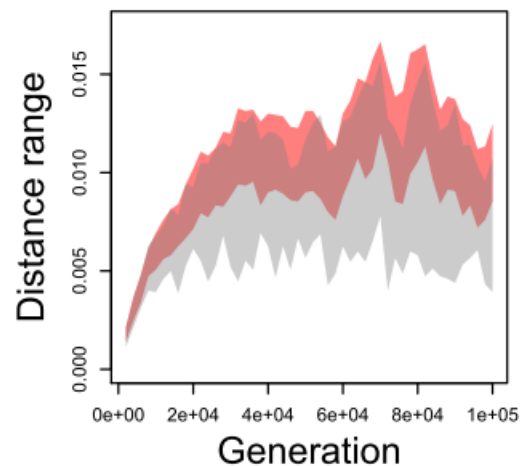**Run= 2****D**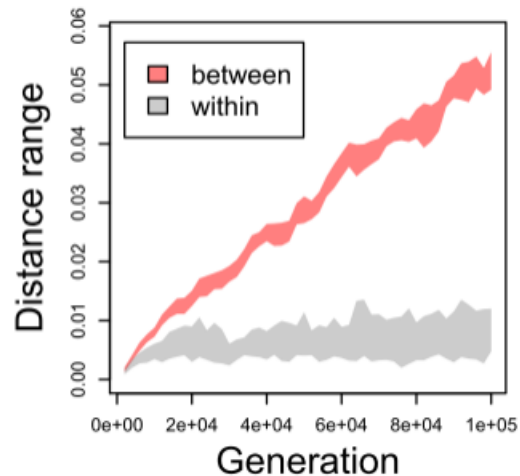**E**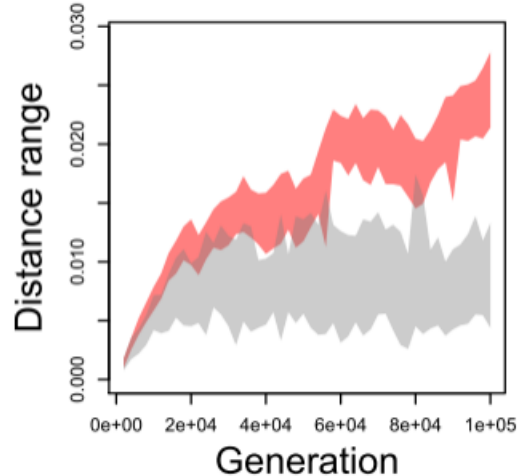**F**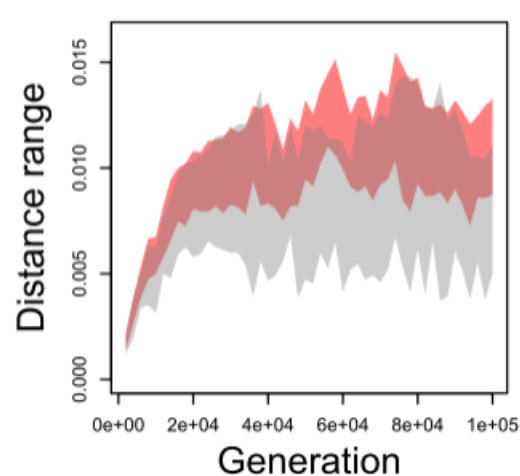**Run= 3**

Supplement: S1 Fig — The figure shows distance range results, interpreted in exactly the same way as Fig 4D–4F in the main text. Rows represent independent simulations of the model, and columns different amounts of habitat overlap. (PDF) [file pcbi.1005640.s002.pdf]

**A** Habitat overlap 20%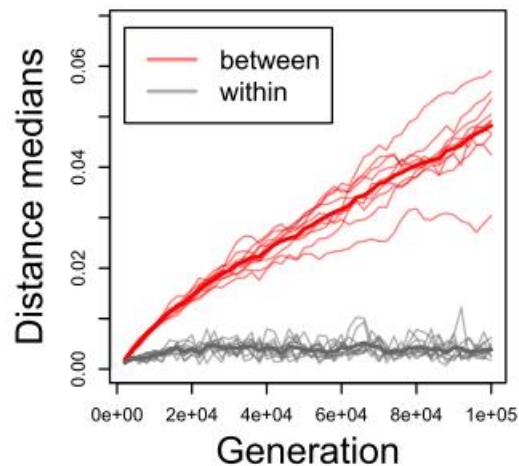**B** Habitat overlap 40%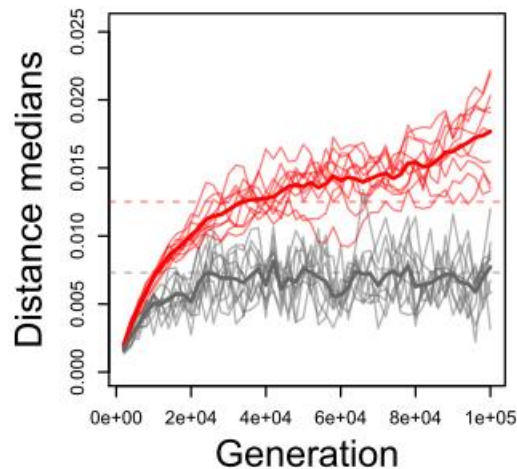**C** Habitat overlap 60%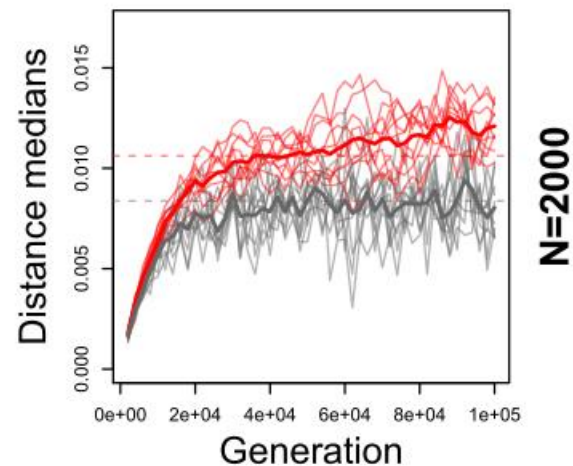**D**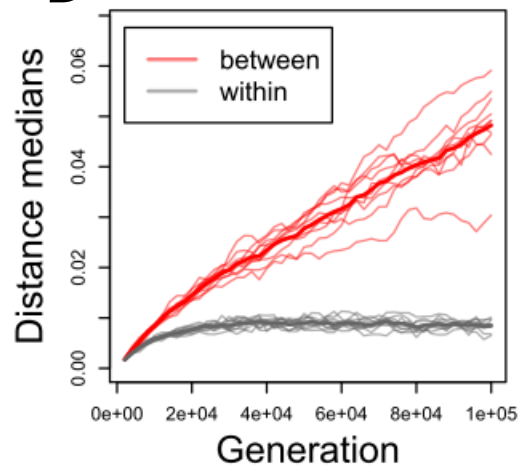**E**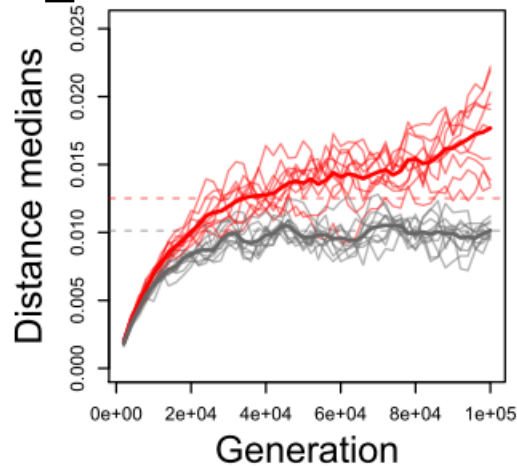**F**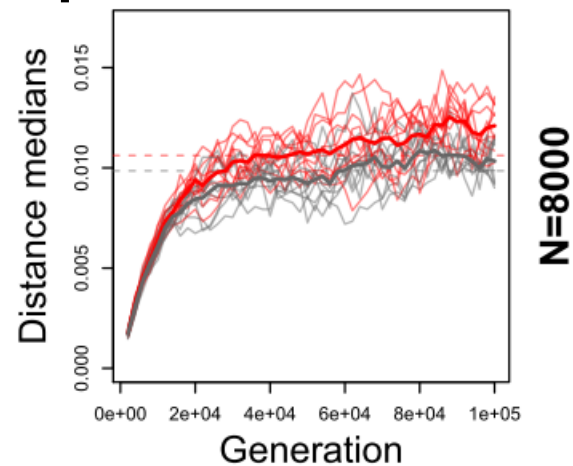

Supplement: S3 Fig — The simulation results in Fig 4 in the main text were based on simulation of 5,000 strains of both types. Here we repeat this with exactly the same parameters, except that 2,000 type A strains and 8,000 type B strains were simulated. The first row shows the within distances in the smaller and the second row in the larger population. The same between distances are shown on both rows. We see that in the larger population there is more diversity than in the smaller one. Nevertheless, the deterministic approximation accurately predicts the within distances in both populations. (PDF) [file pcbi.1005640.s004.pdf]

**Habitat overlap 20%**

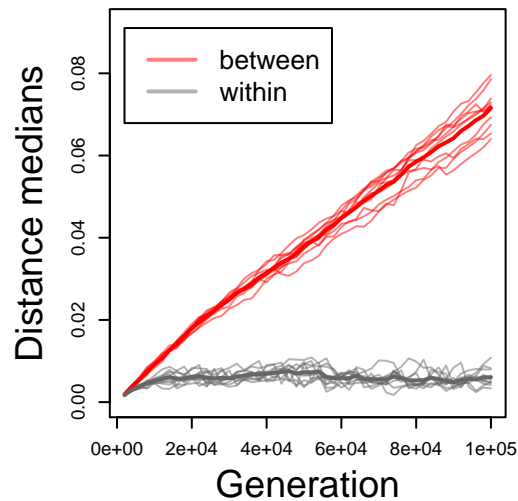

**Habitat overlap 40%**

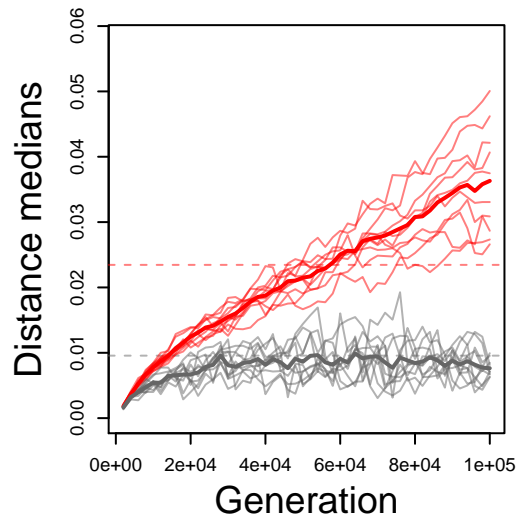

**Habitat overlap 60%**

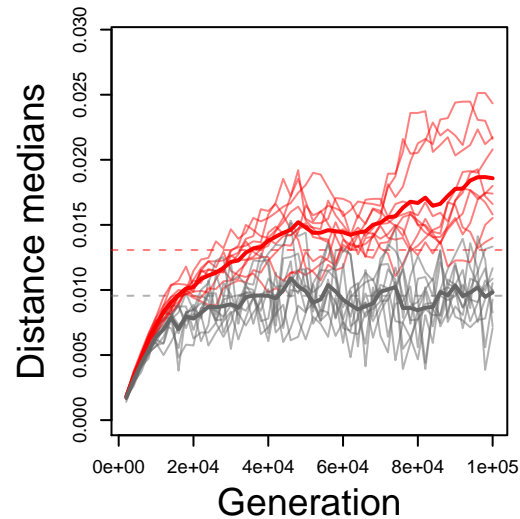

**1/3 times rec**

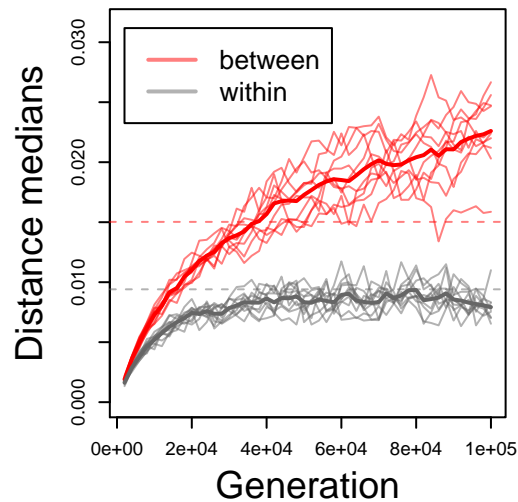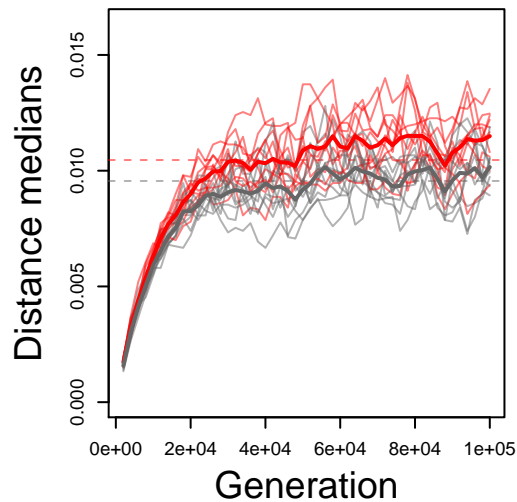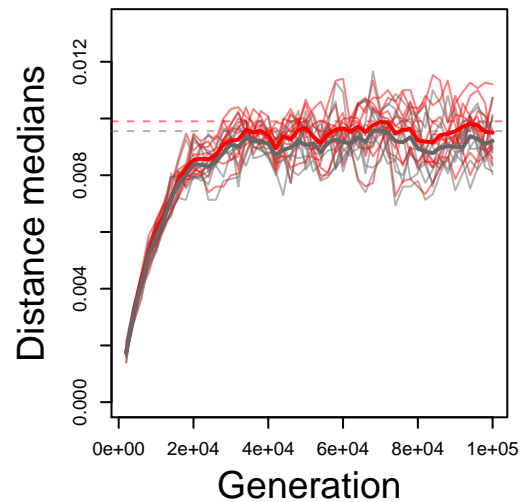

**3 times rec**

Supplement: S4 Fig — The top row shows results with 1/3 and the bottom row 3 times the recombination rate compared to that in Fig 4 in the main text. We see that the between population distance decreases when recombination rate is increased. As has been explained before, the within population equilibrium distance is not affected by the recombination rate, as long as recombination is high enough for the equilibrium to emerge [2, 4]. (PDF) [file pcbi.1005640.s005.pdf]

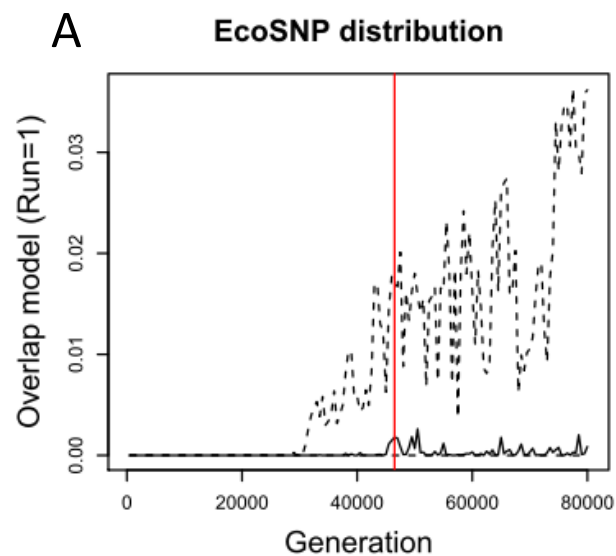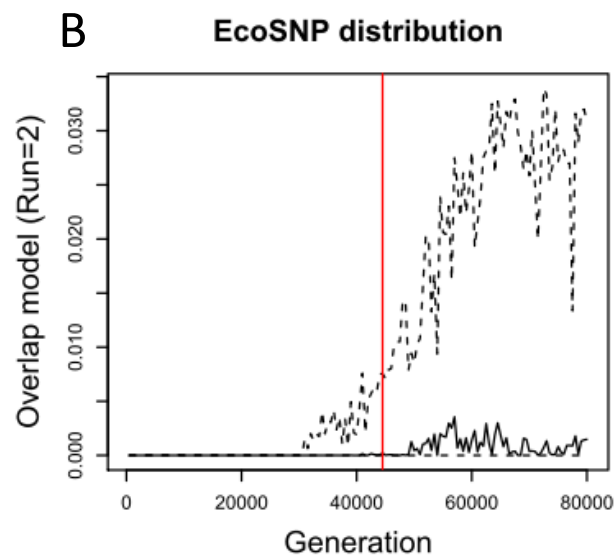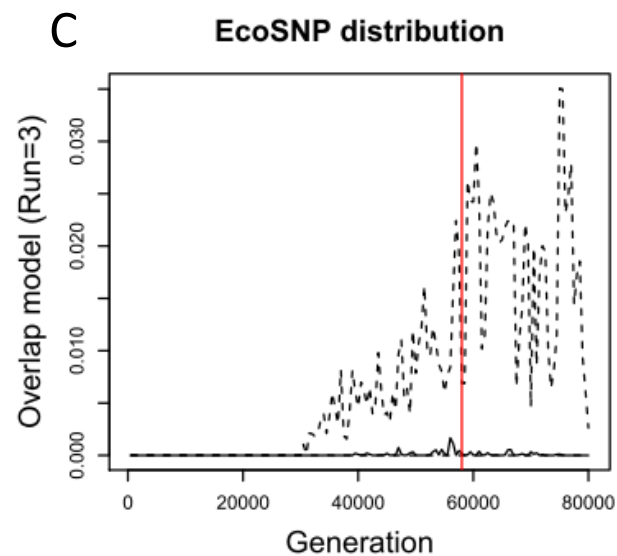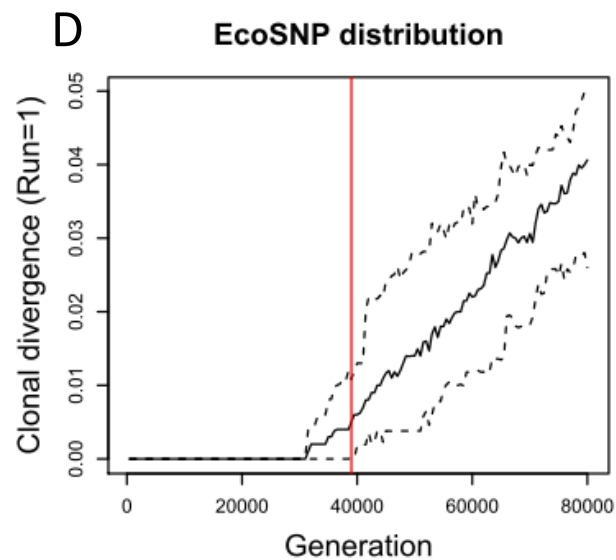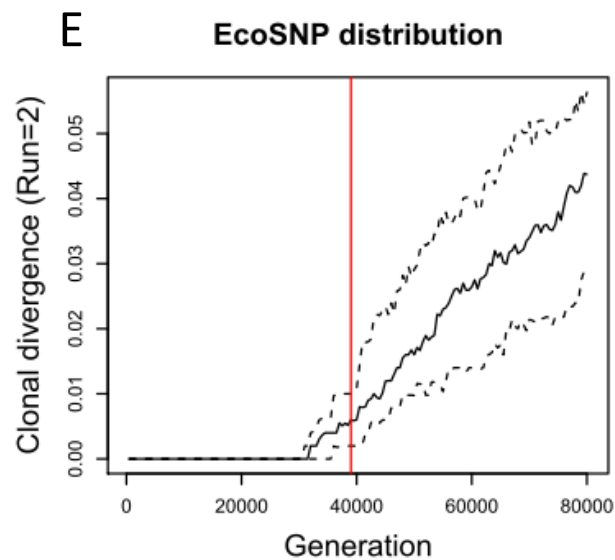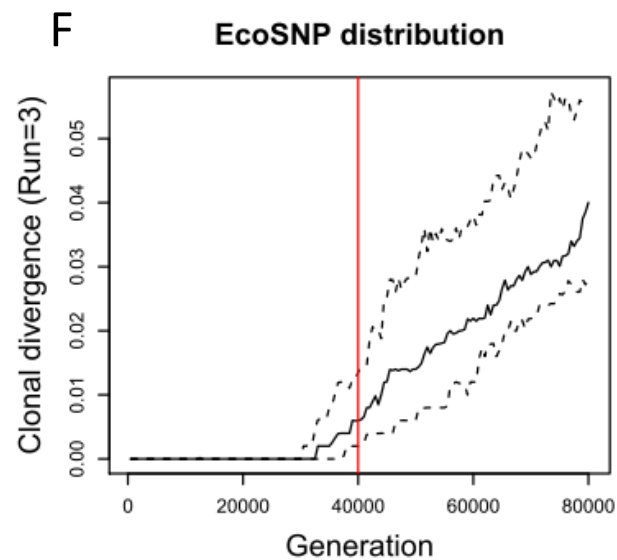

Supplement: S5 Fig — The solid curve shows the median of the ecoSNP distribution, the dashed curves the 0.1th and 0.9th quantiles. The top row corresponds to the simulation from the Overlapping Habitats Model, fitted to the S. pneumoniae data, and the bottom row the corresponding clonal simulation. The colums show results for three independent simulations. The vertical line marks the generation when the between distance matched that observed in the S. pneumoniae data. We see that in the Overlapping Habitats Model (top row) the majority of genes had very few ecoSNPs throughout the simulation, although some genes started to accumulate ecoSNPs immediately after the barrier between the populations had been introduced. In clonal divergence all genes accumulated ecoSNPs at an approximately constant rate. (PDF) [file pcbi.1005640.s006.pdf]

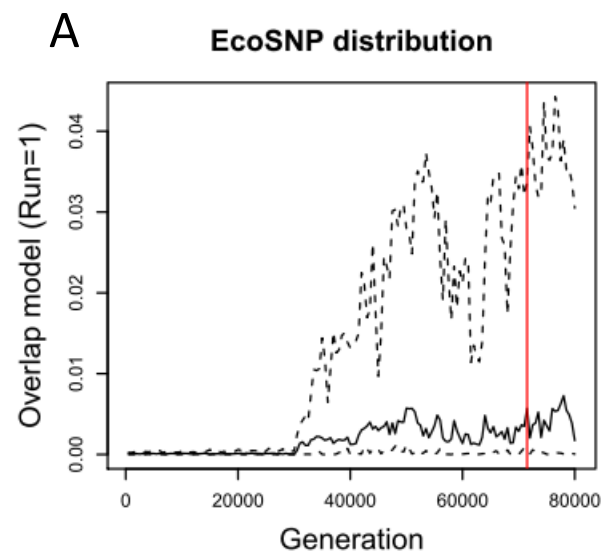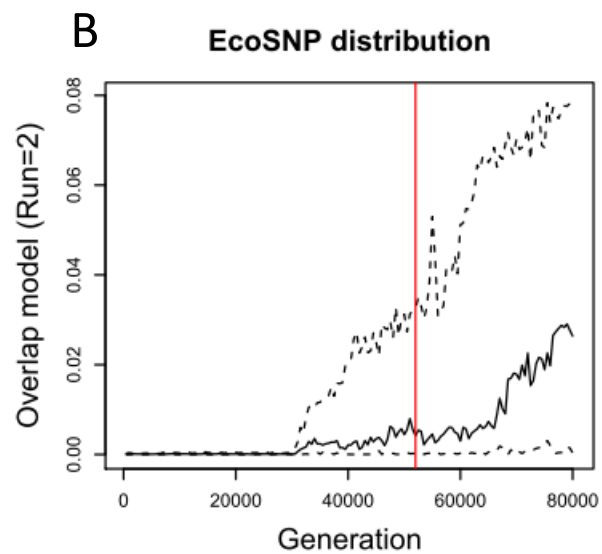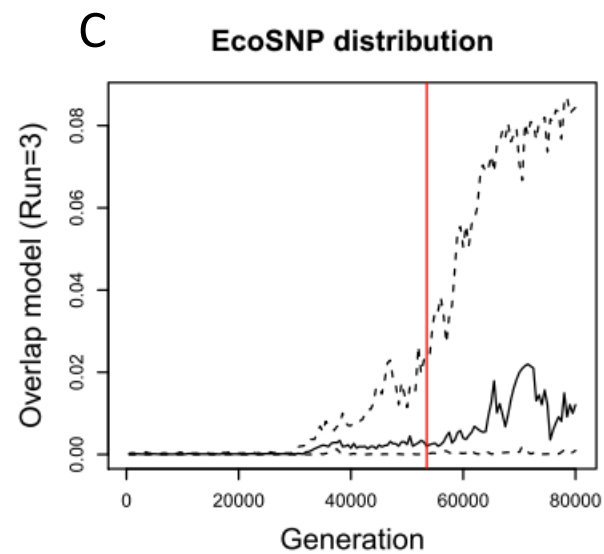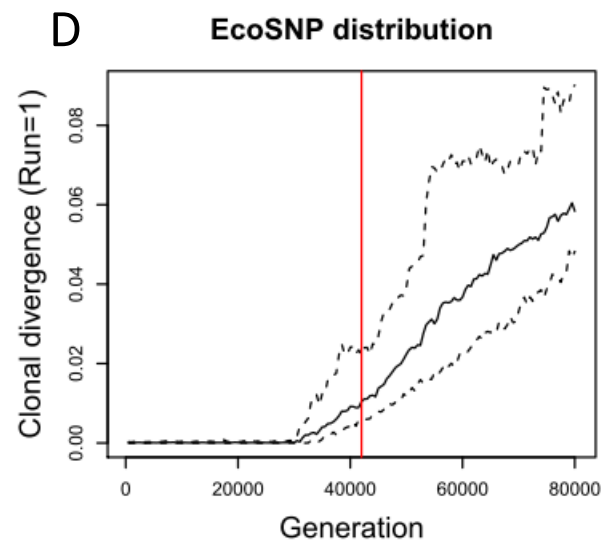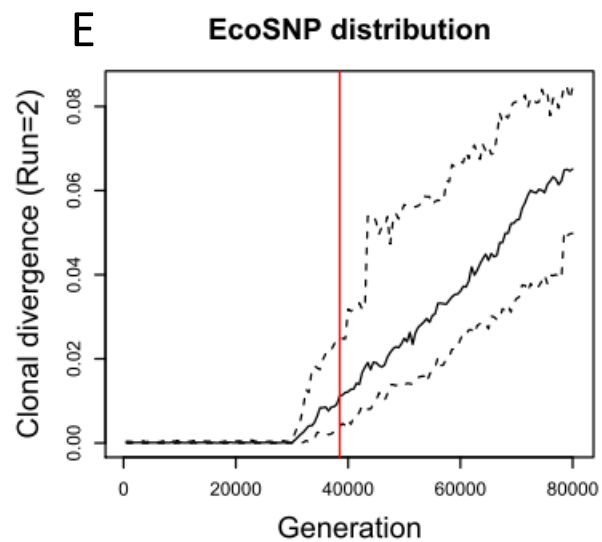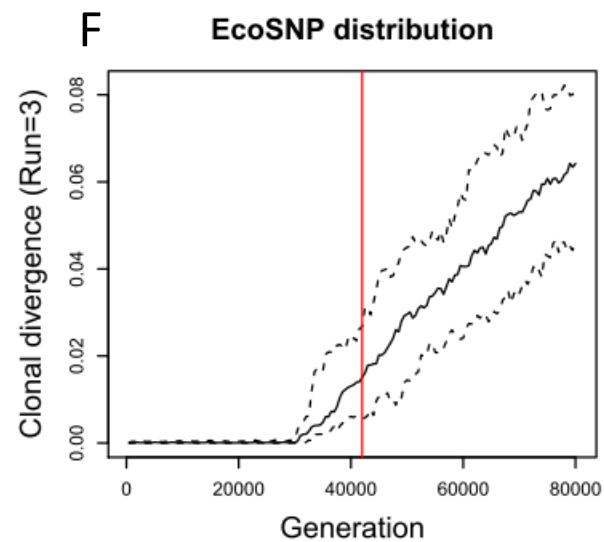

Supplement: S6 Fig — The results are interpreted in the same way as those in S5 Fig. (PDF) [file pcbi.1005640.s007.pdf]

# Divergence rate

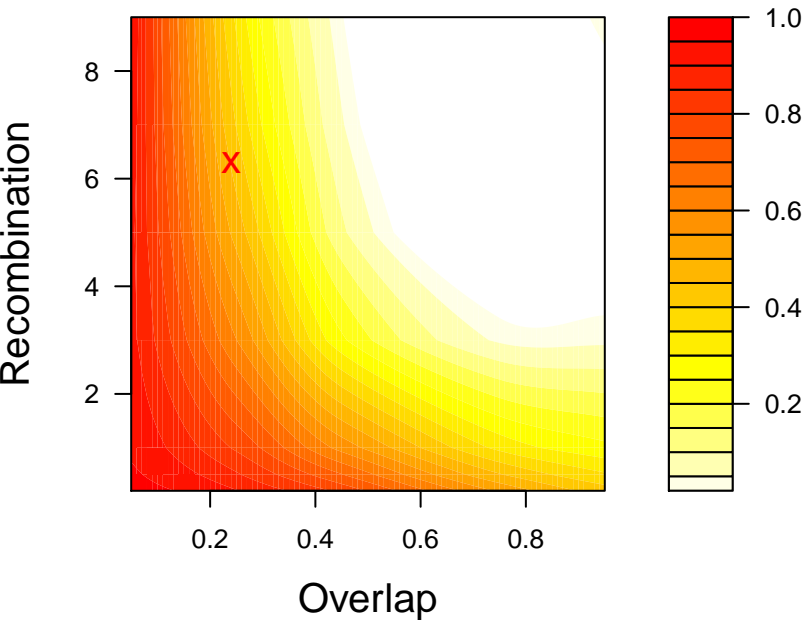

Supplement: S7 Fig — The ‘x’ shows the predicted rate for C. jejuni. (PDF) [file pcbi.1005640.s008.pdf]
