# Supplementary material for: Speciation trajectories in recombining bacterial species
Source: PLoS Comput Biol. 2017 Jul 3;13(7):e1005640. doi: 10.1371/journal.pcbi.1005640 (PMC5542674; doi:10.1371/journal.pcbi.1005640)
Supplement: S2 Fig — Each panel shows median within and between distances in 10 independent simulation runs. Columns represent different amounts of habitat overlap, and rows different migration rates. We see that the results are almost identical for migration≥0.01, and even with migration = 0.001, the results are still qualitatively similar. (PDF) [file pcbi.1005640.s003.pdf]

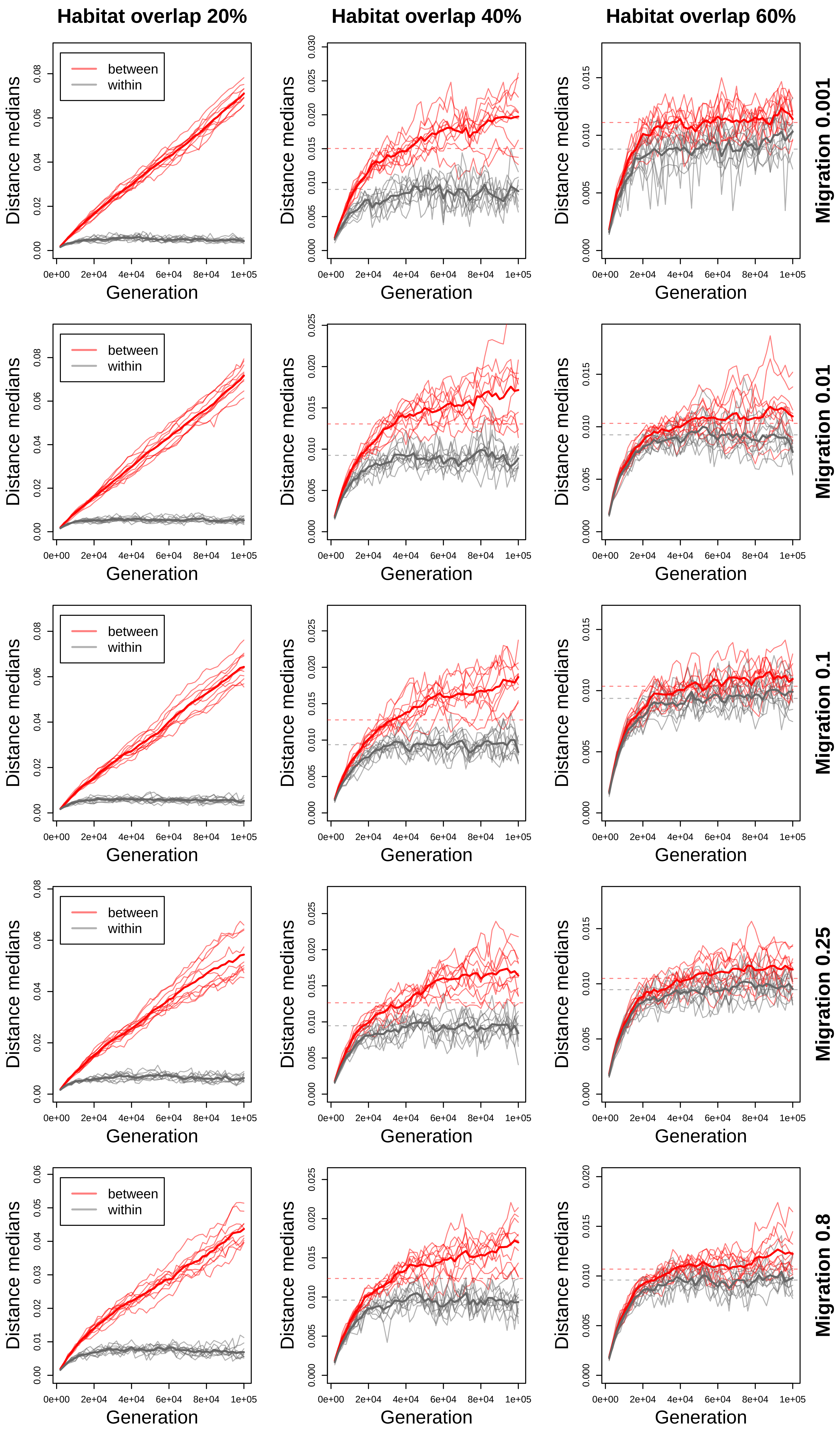
Habitat overlap 20%
Habitat overlap 40%
Habitat overlap 60%
Migration 0.001
Migration 0.01
Migration 0.1
Migration 0.25
Migration 0.8
between
within
Distance medians
Generation
0e+00
2e+04
4e+04
6e+04
8e+04
1e+05
